# Detecting the Influence of Initial Pioneers on Succession at Deep-Sea Vents

**DOI:** 10.1371/journal.pone.0050015

**Published:** 2012-12-04

**Authors:** Lauren S. Mullineaux, Nadine Le Bris, Susan W. Mills, Pauline Henri, Skylar R. Bayer, Richard G. Secrist, Nam Siu

**Affiliations:** 1 Biology Department, Woods Hole Oceanographic Institution, Woods Hole, Massachusetts, United States of America; 2 Université Pierre et Marie Curie, Centre National de la Recherche Scientifique, Banyuls sur Mer, France; 3 Department of Biological Sciences, Virginia Institute of Marine Science, Gloucester Point, Virginia, United States of America; 4 Biology Department, Western Washington University, Bellingham, Washington, United States of America; Heriot-Watt University, United Kingdom

## Abstract

Deep-sea hydrothermal vents are subject to major disturbances that alter the physical and chemical environment and eradicate the resident faunal communities. Vent fields are isolated by uninhabitable deep seafloor, so recolonization via dispersal of planktonic larvae is critical for persistence of populations. We monitored colonization near 9°50′N on the East Pacific Rise following a catastrophic eruption in order to address questions of the relative contributions of pioneer colonists and environmental change to variation in species composition, and the role of pioneers at the disturbed site in altering community structure elsewhere in the region. Pioneer colonists included two gastropod species: *Ctenopelta porifera*, which was new to the vent field, and *Lepetodrilus tevnianus*, which had been rare before the eruption but persisted in high abundance afterward, delaying and possibly out-competing the ubiquitous pre-eruption congener *L. elevatus*. A decrease in abundance of *C. porifera* over time, and the arrival of later species, corresponded to a decrease in vent fluid flow and in the sulfide to temperature ratio. For some species these successional changes were likely due to habitat requirements, but other species persisted (*L. tevnianus*) or arrived (*L. elevatus*) in patterns unrelated to their habitat preferences. After two years, disturbed communities had started to resemble pre-eruption ones, but were lower in diversity. When compared to a prior (1991) eruption, the succession of foundation species (tubeworms and mussels) appeared to be delayed, even though habitat chemistry became similar to the pre-eruption state more quickly. Surprisingly, a nearby community that had not been disturbed by the eruption was invaded by the pioneers, possibly after they became established in the disturbed vents. These results indicate that the post-eruption arrival of species from remote locales had a strong and persistent effect on communities at both disturbed and undisturbed vents.

## Introduction

Deep-sea hydrothermal vents experience major disturbances that eradicate the resident faunal communities and alter the physical and chemical environment. Prior studies have documented faunal successional changes after an eruptive disturbance [1]–[4]. These changes often correlate with changes in vent fluid flow rate and composition and subsequent environmental changes. Vent fluid sulfide-to-temperature ratios tend to decrease, over years after an eruption [5], leading to an increase in oxygen and decrease in sulfide in faunal habitats, and potentially to lower microbial production and reduced toxicity levels. Biological interactions, however, are also known to affect succession at vents [6]–[8]. In particular, species composition of pioneers may play an important role [9]. If succession at vents follows a deterministic trajectory set by species’ response to decreasing temperature and sulfide levels, as suggested in Shank et al. [3] and Marcus et al. [2], then response of the community to future disturbances should be predictable based on environmental conditions. If, however, succession is influenced by availability of particular pioneer colonists at the time of the disturbance, then initial conditions need to be considered.

The occurrence of alternative successional trajectories has been documented for terrestrial, and more recently, coastal marine systems (reviewed in [10], [11]). The initial conditions in these systems, such as availability of propagules, and presence of predators and competitors at the time of disturbance, are especially important. These conditions may ‘canalize’ succession and lead to quite different outcomes [12]. Determining whether this process occurs at deep-sea vents is important in order to predict how vent communities will respond to future natural and human disturbance. On the East Pacific Rise, results from a prior (1991) eruption and from manipulative experimentation have suggested that the siboglinid (vestimentiferan) tubeworm *Tevnia jerichonana* is a pioneer colonist, followed in succession by the larger, faster-growing tubeworm *Riftia pachyptila*, and then the mussel *Bathymodiolus thermophilus* [1], [3], [8]. This sequence may or may not be typical, and the roles of other numerous and diverse groups, such as gastropods, remain largely unexplored.

The transient nature of vents, and their patchy communities connected by larval dispersal, make them well suited to a metapopulation approach [13]. Theoretical considerations have suggested that in metapopulations where vents expire and re-open at different locations, individual populations rarely experience extinction, and population genetic structure becomes homogeneous [14]. This model, however, did not incorporate variation in larval supply or biological interactions during succession, which have a substantial influence on diversity in newly colonized vents [13]. An understanding of the processes that control succession is critical in order to answer questions about how regional species diversity is affected by natural or anthropogenic disturbance.

A catastrophic seafloor eruption near 9°50′N on the East Pacific Rise (EPR) provided an opportunity in 2006 to monitor faunal succession at vents after the regional faunal communities were destroyed. A rapid response to the eruption provided information on pioneer colonization within six months of the event. Faunal observations at multiple sites coupled with detailed in situ measurements of seafloor habitat allowed documentation of changes in species composition in the context of variation in the physical and chemical environment. Our main goal was to characterize the pioneer colonists and determine whether and how they influenced the trajectory of succession in this dynamic environment. We monitored colonization over time, and used comparisons within and between selected vent sites to assess the effects of disturbance history (i.e., perturbed and unperturbed sites), and document correlations between faunal abundance and habitat conditions, in order to address the following specific questions:

1. How does colonization vary over the two-year period following the eruption?
2. What are the relative contributions of pioneer colonists and environmental change to variation in species composition?
3. How does pioneer colonization at a disturbed site influence community structure at sites elsewhere in the region?

## Methods

### Ethics Statement

Field sampling and experimentation at the vent sites was conducted according to guidelines recommended for international waters by InterRidge in their statement of commitment to responsible research practices at deep-sea hydrothermal vents. No specific permits were required for the described field studies. No specific permissions were required because the sites are in international waters. The location is not privately owned or protected in any way. The field studies did not involve endangered or protected species.

**Figure 1:**
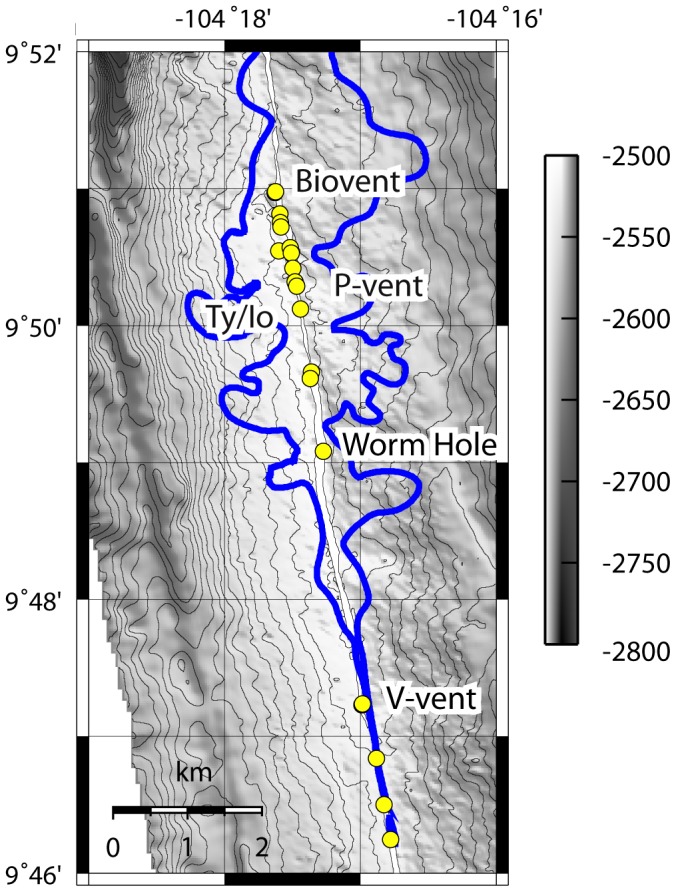
Map of vent locations on ridge axis of East Pacific Rise near 9°50′N. Vents (yellow circles) referenced in text are named. Blue outline shows extent of lava from 2006 eruption.

Biological collections and chemical measurements were conducted on a series of post-eruption cruises to the 9°50′N region of the EPR between July 2006 and January 2008 (Table 1). Temporal monitoring focused on P-vent (Fig. 1), a site that had been covered by lava in the eruption. Colonization studies were initiated 5 months after the January 2006 eruption, and monitored at 9, 11 and 22 months. Habitat chemistry was measured at 11 and 23 months. The Ty/Io vent was visited at 5 and 9 months post-eruption, but over that interval, temperatures in the diffuse-flow habitat (near Marker 8) declined precipitously, so experiments were discontinued there. Disturbance effects were investigated by comparing the final (22-month) observations from P-vent to those from concurrent deployments at nearby V-vent, which had not been disturbed, and to pre-eruption observations [15] from neighboring sites Biovent and Worm Hole (Fig. 1). Environmental effects were assessed at P-vent at 11 months after the eruption in habitats characterized as hot, warm or cool, corresponding to the vestimentiferan, bivalve and suspension-feeder zones of Micheli et al. [7]. Hot habitat was characterized by the presence of tubeworms, vigorous vent fluid flow, and maximum temperatures up to 30°C. Warm habitat lacked tubeworms and had moderate vent fluid flow with temperatures <7°C, and cool habitat was characterized by sparse fauna, weak vent fluid flow, and temperatures <4°C. Investigation of temporal change and disturbance effects focused on hot habitat.

**Table 1:** Environmental conditions measured at colonization surfaces or on seafloor.

Object	Time (mo)	Site	Habitat	Measure	N
Sandwich	9	P	H	T	3
		Ty/Io	W	T	3
	11	P	H, W, C	T	3
		V	Hot	T	3
	22	P	H, W, C	T	3
		V	H, W, C	T	3
Block	Pre	BV	H	T	4
		WH	H	T	2
Seafloor	11	P	H	T, pH, sul	6
			C	T, pH, sul	3
		V	H	T, pH, sul	6
			W	T, pH, sul	6
	23	P	H	T, pH, sul	6

Measurements recorded at 9, 11, 22 or 23 months after the eruption, during cruises in October 2006, December 2006, and November/December 2007 respectively, and pre-eruption (Pre) in December 1995. Sites were vents in the region of 9°50′N East Pacific Rise (Fig. 1), habitats were characterized as hot (H), warm (W) or cool (C) on initial visit. Temperature (T) was measured directly at colonization surfaces with Alvin probe. Temperature, pH and free sulfide (sul) were measured at the seafloor with electrochemical sensors in the vicinity of colonization surfaces and at other nearby vents (N = number of measurements in each habitat type).

Colonists were collected on experimental surfaces (sandwiches) constructed from six 0.7-cm-thick Lexan plastic plates separated by 1 cm spacers, creating a lattice 10 cm on a side [16]. Sandwiches were deployed and recovered by the submersible Alvin. Deployment durations were 4, 2, and 11 months respectively for the recoveries at 9, 11, and 22 months after the eruption. The thermal environment at the base of each sandwich was measured with the Alvin temperature probe on deployment and recovery for ca 1–2 min until a clear maximum value was obtained. The maximum value of the recovery temperature was used to characterize the thermal environment of the surface, for consistency with prior studies (e.g., [15]). Time-series temperature recorders (Hobos) were placed near colonization surfaces in each habitat at P-vent (9 to 11 and 11 to 22 months post eruption) and V-vent (11 to 22 months), recording at 4 or 6 measurements per second, depending on deployment duration.

Species abundance was assessed on three replicate sandwiches for each treatment (e.g., from one habitat within a site at a particular recovery date). On recovery, each sandwich was placed in individual collection compartments for transport back to the ship. On board, sandwiches and their attached colonists were preserved in 80% ethanol, as were any individuals that had become detached in the compartment and were retained on a 63-µm sieve. In the laboratory, each surface was examined under a dissecting microscope and all metazoan colonists (including detached individuals >1 mm) were enumerated and identified to species if possible. Data from the colonization experiments have been deposited in the Ridge2000 data base as part of the Marine Geoscience Data System at Lamont Doherty Earth Observatory of Columbia University (http://www.ldeo.columbia.edu/research/marine-geology-geophysics/mgds-ridge-2000-data-portal).

In the pre-eruption study [15], colonization had been assessed on basalt blocks, 10 cm on a side, deployed for a 13-month interval (Nov. 1994 to Dec. 1995) in the hot habitat at Biovent and Worm Hole. This combination of site and deployment interval was selected to match as closely as possible the environment and duration of the final post-eruption deployment. We assume that pre-eruption communities at P-vent and V-vent were similar to those at Biovent and Worm Hole, based on observations of faunal similarity on these spatial scales in the 9°50′N region [3], [15], [17], but cannot discount the possibility of minor site-related faunal variation. Colonists from sandwiches and blocks were processed and identified in the same way. Species abundance on blocks and sandwiches were not directly comparable because the sandwiches have greater surface area, but a simultaneous deployment of the two surface types demonstrated no significant difference in relative species abundances between the two [9]. Therefore, comparisons of colonization between blocks and sandwiches were conducted with relative, rather than absolute, species abundance. Although the experimental surfaces do not mimic exactly natural substratum, our prior studies [9], [15], [18] have demonstrated that they collect nearly all species reported from natural habitat in similar fluid environments [19], [20] and no unusual species not reported by taxonomists for this region [21].

To characterize the vent habitats, two main chemical parameters were measured: free sulfide (HS^−^ + H_2_S), representing the main energy source for vent communities [22], and pH, a tracer of the fluid subsurface transformations also controlling sulfide toxicity [23]. Electrochemical sensors were positioned in the immediate vicinity of colonization surfaces at P-vent and V-vent to assess chemical variation at the scale of the deployment area. At 11 months post-eruption these measurements were obtained in hot and cool habitat at P-vent, and hot, warm and cool habitat at V-vent. At 23 months, they were obtained only in hot habitat at P-vent. The pH and free sulfide electrode tips (respectively 1.5 and 0.8 µm in diameter) were combined together and tightly attached to a temperature sensor (3 mm diameter). Sensor responses and temperature were recorded over ca 1 to 3 minutes on selected locations at a frequency of once every 5 seconds. The records at any one location integrate both natural fluctuations (turbulence) and, in some cases, slight movements of the probe (less than a few cm as assessed from video observation). For each habitat within a site, data were acquired from 3 to 6 different locations to define the range in environmental conditions (Table 2, Table S1). The maximum temperature, maximum sulfide and minimum pH of multiple scans were used to calculate mean values for comparison with colonization studies.

**Table 2:** Temperature (T°C) and chemical measurements (pH and free sulfide) analyzed by site.

		P Vent			V Vent		
		11 mo		23 mo	11 mo		
Habitat		Hot	Cool	Hot	Hot	Warm	Cool
No. scans		6	3	6	6	6	2
No. data		157	96	66	54	163	87
T °C	Min	10.3	2.4	2.1	2.3	2.4	1.8
	Med	16.8	3.2	9.5	5.5	2.9	1.9
	Mean	19.7	3.1	8.5	9.1	3.6	1.9
	Max	29.7	4.0	14.0	19.2	7.5	2.1
	Stdev	6.8	0.5	0.5	6.1	1.5	0.1
pH	Min	5.8	7.1	6.1	6.6	7.2	7.9
	Med	6.3	7.7	6.4	7.3	7.7	7.9
	Mean	6.2	7.6	6.6	7.2	7.7	7.9
	Max	6.8	8.0	7.6	7.7	7.9	7.9
	Stdev	0.3	0.2	0.5	0.3	0.2	0.0
Sulf µM	Min	39	0	0	1	0	0
	Med	210	1	16	34	3	0
	Mean	295	2	23	83	6	0
	Max	765	7	115	297	36	0
	Stdev	208	2	29	89	8	0

Habitat is Hot, Warm or Cool; time is months from 2006 eruption. Number of scans are individual measurements taken at different locations within each site. Number of data is total number of measurements pooled over scans. Statistics include minimum, median, mean, maximum and standard deviation.

Analyses at the community level were conducted with nonmetric multidimensional scaling (nMDS; Systat v. 13) to visualize the similarity in species abundance in comparisons of time, disturbance and environment. A principal components analysis (Systat v. 13, varimax rotation, using a Pearson’s correlation matrix) was also used to explore the extent of correlation in species abundance between samples. Numbers of colonists varied widely between treatments, so a rarefaction analysis (BioDiversity Pro, with total individuals pooled over replicates) was used to compare species diversity. For the comparisons of time, disturbance, and habitat, analyses of selected individual species were conducted with univariate ANOVA. Included in these analyses were the initial pioneers (those in the 9-month recovery in hot habitat at P-vent with mean abundance >1/sample) and additional species (up to 6 total) identified as important in previous successional studies (e.g., *Riftia pachyptila, Bathymodiolus thermophilus* in Shank et al. [3]) or as abundant in later recoveries or at other sites. In comparisons of time and disturbance, abundance values were calculated relative to total number of individuals in sample (to compensate for different deployment intervals or surface types), and transformed (arcsine square root) to approximate normality and homogeneity of variance. In comparison between habitats, raw abundance was used because deployment interval and surface type were equivalent across samples. These values were log-transformed to approach homogeneity of variance. Significance was assessed with a Bonferroni correction for multiple tests at a level corresponding to P<0.05 (MANOVA would have avoided the need for the correction, but could not be used because the number of species exceeded the number of replicates). In tests with more than two treatment conditions, significant differences between conditions were identified with a post-hoc Tukey test.

Faunal differences over time, between disturbance histories, and between habitats were compared qualitatively to corresponding variation in chemical habitat. Direct comparison via regression or other statistical methods was not attempted because chemical sensors measured heterogeneity on scales of mm to cm, whereas colonization results integrated over the 10-cm scale of sandwiches and blocks. Furthermore, the chemical probes were not necessarily positioned directly under sandwiches, as was the Alvin temperature probe. Nevertheless, by establishing a correlation between temperature and sulfide or pH, habitat conditions can still be compared independently of the local variability of the fluid dilution ratio [24], and temperature can be used to estimate the chemical environment of colonization experiments.

## Results

### Post-eruption Environment

**Figure 2:**
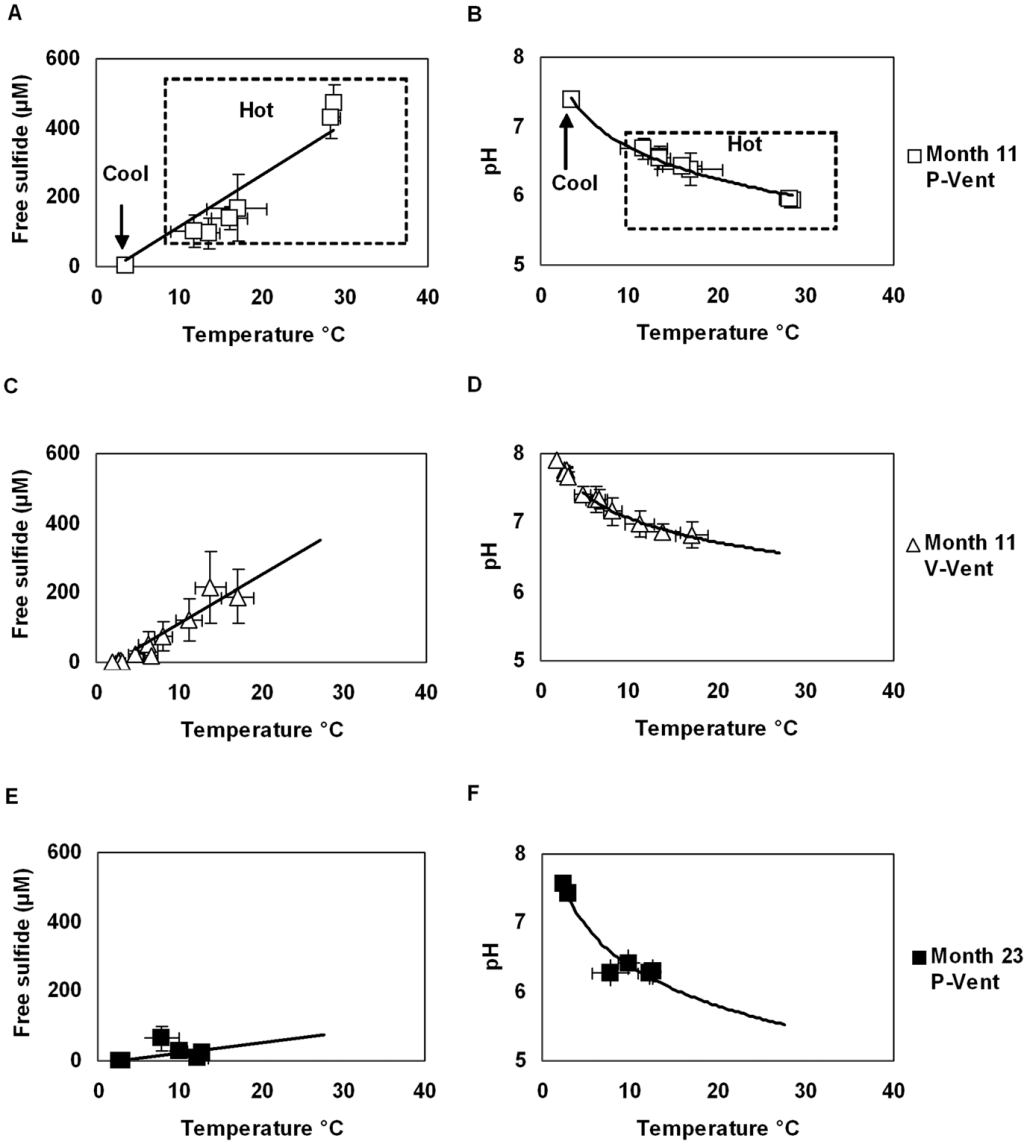
Variability of habitat conditions at P-vent and V-vent at 11 and 23 months after eruption. Mean and standard deviation are displayed for each measurement location. Free sulfide and pH are displayed as function of temperature, together with the corresponding regression curve (linear and logarithmic models respectively). Curves were extrapolated to 30°C in C–F for comparison. (A, B) hot and cool habitats at 11 months at P-vent; (C, D) hot and cool habitats at 11 months at V-Vent; (E, F) hot habitat at 23 months at P vent. Statistics for individual scans are in Table 2; statistics pooled for sites are in Table S1.

Fluid habitat measurements taken at P-vent at 11 months after the eruption show a linear increase of free sulfide with temperature, and a logarithmic decrease of pH (Fig. 2A, B). These same general patterns were observed at V-vent (Fig. 2C, D) and in multiple nearby tubeworm-inhabited vent sites at that time (Le Bris, unpublished data). At P-Vent at 23 months after the eruption, a deviation from this trend was observed (Fig. 2 E, F). Standard deviations for 1 to 3-min measurement periods were substantial for sulfide (Fig. 2, Table S1). This variation is not unexpected given the spatial and temporal heterogeneity of vent habitats, although it is partly amplified by measurement uncertainty at low pH. Fluctuating conditions reflect filaments of vent fluid forming vortices while mixing with colder seawater as they exit the seafloor. It is important to recognize that short-term (<3-min) measurements do not necessarily characterize the variation of conditions experienced over semidiurnal and longer tidal periods at a single location. However, the longer-term variance in temperature, as recorded by Hobo loggers over the scale of months (Fig. S1), was within the range of spatial variation documented in point measurements. Thus, the scans appear to capture the full range of environmental conditions at each site even though they don’t represent the long-term variation at any one particular point.

The consistent correlation between temperature and chemical characteristics at P vent at 11 months (Fig. 2A, B) suggests that the environmental conditions experienced by organisms in the experiment area were primarily controlled by the dilution of the local vent fluids by ambient seawater, as previously described [5], [24]. The relation between free sulfide or pH and temperature in habitats appears to be similar between P and V vent sites (Fig. 2A–D) and, concerning sulfide, unaltered by the resident organisms. This consistent correlation means that colonization surfaces that experienced similar temperatures at 11 months probably also experienced similar chemical conditions. These relationships changed significantly over time at P vent, with a large decrease in the sulfide to temperature ratio between 11 and 23 months (Fig. 2E). The pH to temperature relation also shifted toward lower pH for a given temperature range (Fig. 2F). These variations indicate changes in the source fluid properties which appear depleted in sulfide and more acidic, a change which is likely due to subsurface biotic and abiotic processes (e.g. iron sulfide precipitation both leading to free sulfide consumption and acidification).

### Temporal Change

**Figure 3:**
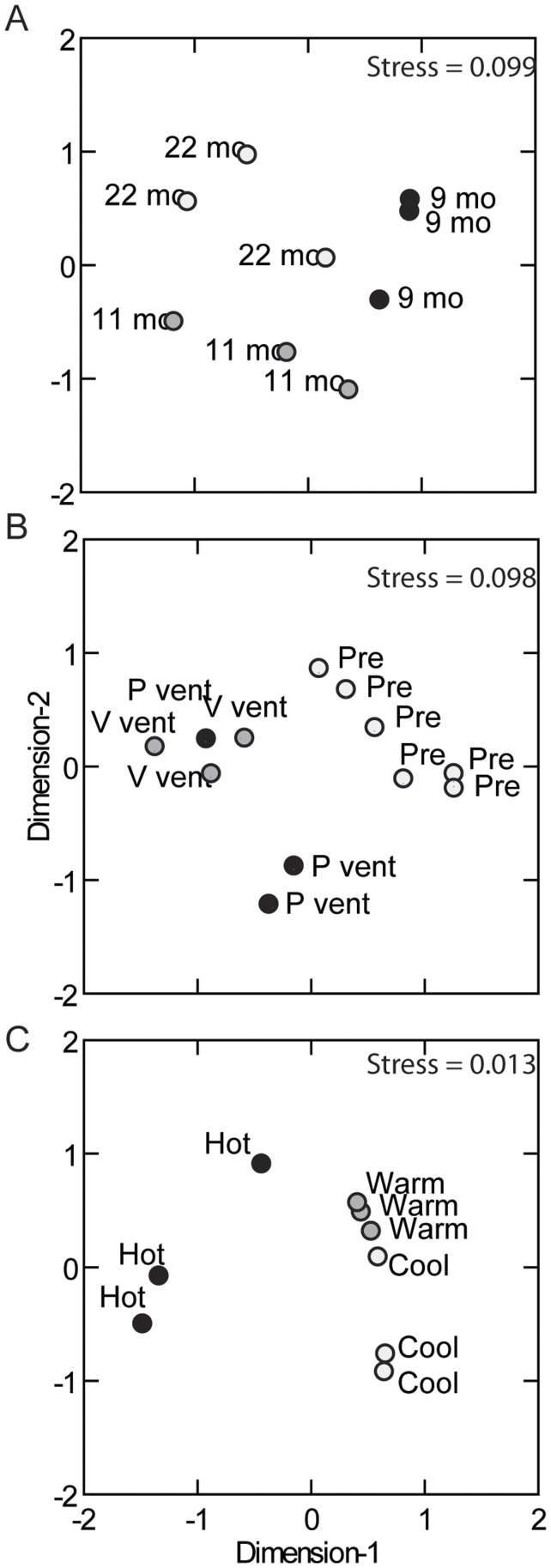
Non-metric multidimensional scaling analysis (nMDS) of colonist species composition. Closely clustered symbols correspond to samples with similar species composition and abundance. Comparisons are made: (A) across Time (9, 11 and 22 months after eruption at P-vent hot habitat); (B) between Disturbance history (pre-eruption at Biovent and Worm Hole hot habitat, and post-eruption at P-vent and V-vent hot habitat); and (C) between Habitat (hot, warm and cool habitat at P-vent, 11 months post-eruption). Stress values near zero indicate that most of the variance in species abundance was accounted for in the analysis.

Over 50 different species and species groups were identified from the combined pre- and post-eruption colonization samples (Table 3, Table S2). Community-level analysis (nMDS) of colonization in P-vent hot habitat at 9, 11 and 22 months post-eruption showed a general pattern of change in species composition over time, although variation between replicates was high and samples did not segregate into discrete clusters (Fig. 3A). This variation between replicates within each recovery time is consistent with the high spatial variability of physico-chemical conditions in these habitats. Results of principal components analysis were similar to nMDS and are not shown.

**Table 3:** Abundance of species (taxa) on colonization surfaces at different sites, times and environments.

		P-vent					Ty/Io	V-vent	Biovent	WH
		9 mo	11 mo			22 mo	9 mo	22 mo	Pre	Pre
Group	Taxon	Hot	Hot	Warm	Cool	Hot	Warm	Hot	Hot	Hot
Mollusca	*Bathymargarites symplector*	0	0	0	0	0	0	0	0	1
	*Clypeosectus delectus*	0	0	0	10	0	4	0	0	1
	*Ctenopelta porifera*	75	46	0	0	16	1	3	0	0
	*Cyathermia naticoides*	8	9	0	3	1363	1	121	180	27
	*Eulepetopsis vitrea*	0	0	0	1	0	2	0	0	0
	*Gorgoleptis spiralis*	0	0	0	8	0	3	0	3	0
	*Gorgoleptis emarginatus*	0	0	3	0	0	0	0	0	0
	*Lepetodrilus cristatus*	0	0	0	0	0	0	0	27	0
	*Lepetodrilus elevatus*	0	0	2	0	716	2	74	835	2632
	*Lepetodrilus ovalis*	0	0	3	0	1	0	1	1	2
	*Lepetodrilus pustulosus*	0	0	14	0	1	0	0	56	93
	*Lepetodrilus tevnianus*	22	71	2163	640	719	3203	260	0	0
	*Lepetodrilus* spp.	1	0	1001	1260	298	3427	30	0	0
	*Pachydermia laevis*	0	0	0	0	0	2	0	0	0
	*Planorbidella planispira*	0	0	0	0	0	1	0	0	0
	*Rhynchopelta concentrica*	0	0	0	0	5	1	0	3	0
	*Sutilizona theca*	0	0	0	6	0	9	0	0	0
	gastropods, unk	0	0	6	0	0	0	0	68	10
	*Bathymodiolus thermophilus*	0	0	1	1	4	2	3	38	13
	aplacophoran	0	0	0	0	0	0	0	1	1
Polychaeta	*Alvinella* sp.	1	0	0	0	0	0	0	0	0
	*Amphisamytha galapagensis*	0	0	39	4	13	123	3	378	55
	*Archinome rosacea*	0	0	1	0	0	0	0	0	0
	*Branchinotogluma* sp.	0	0	0	0	3	0	3	0	0
	*Branchiplicatus cupreus*	0	0	0	0	5	0	4	0	0
	*Branchipolynoe* sp.	0	0	0	0	0	0	0	0	1
	*Galapagomystides aristata*	0	0	3	2	0	0	0	26	0
	*Glycera* sp	0	0	0	1	0	0	0	0	0
	*Hesiospina vestimentifera*	0	0	0	0	1	0	0	0	0
	hesionid	0	0	0	10	0	0	0	0	0
	*Laminatubus alvini*	0	0	0	0	0	0	0	9	1
	*Lepidonotopodium* sp.	0	2	0	0	0	0	1	0	1
	*Nereis* sp.	0	0	0	0	0	0	0	1	0
	*Ophryotrocha akessoni*	0	0	58	66	313	164	98	71	16
	*Paralvinella grasslei*	391	238	1	1	338	5	49	51	0
	polynoids, unk	0	1	9	31	0	5	1	2	0
	*Prionospio sandersi*	0	0	0	0	1	0	0	0	0
	polychaetes, unk	0	1	3	0	0	139	0	1	0
	*Riftia pachyptila*	0	0	0	0	2	0	0	6	3
	*Tevnia jerichonana*	0	0	0	0	261	0	122	10	3
	vestimentiferans, small	1996	39	48	0	897	1554	847	661	957
Crustacea	amphipod	2	35	107	8	1389	5	1537	52	32
	*Bythograea thermydron*	8	7	4	0	5	1	7	0	0
	*Dahlella caldariensis*	0	0	0	0	0	0	29	0	4
	juv. shrimp	0	0	0	0	0	1	0	0	0
Other	unsegmented worm	0	0	2	0	34	1	5	0	4
	anemone	0	0	0	0	0	1	0	0	0
	dandelion	1	0	0	0	0	0	0	0	0
	kinorhynch	0	0	0	0	0	3	0	0	0
	*Abyssotherma pacifica*	0	0	0	0	0	0	0	3	2
	*Metafolliculina* sp.	0	0	0	0	0	0	0	0	3

Values pooled from replicate surfaces, n = 3 except for Biovent (n = 4) and Worm Hole (WH; n = 2). Sandwiches recovered from hot, warm or cool environment at times 9, 11 or 22 months after the eruption at P-vent, Ty/Io, and V-vent. Blocks recovered from hot habitat before the eruption (Pre) at Biovent and Worm Hole. Surface areas of blocks and sandwiches were 600 cm^2^ and 1200 cm^2^, respectively.

Examination of individual species abundance provides additional detail on temporal patterns in community structure.One complication for the species-level successional analysis was the difficulty in identifying small siboglinid tubeworms to species. In the recovery at 22 months, individuals had grown large enough to identify as *Tevnia jerichonana* or *Riftia pachyptila*, but in shorter term deployments many of the tubeworms were too small to identify. Given that *T. jerichonana* was the overwhelming dominant among identified individuals in our samples (only two *R. pachyptila* were found) and in the surrounding community (only occasional isolated individuals of *R. pachyptila* were observed by authors at P-vent and other nascent sites), we chose to categorize small unidentified siboglinid tubeworms as *T. jerichonana* in the temporal analysis. Species identification was not a problem in comparisons between disturbance conditions, as tubeworms were included in the analysis only when identified to species.

**Figure 4:**
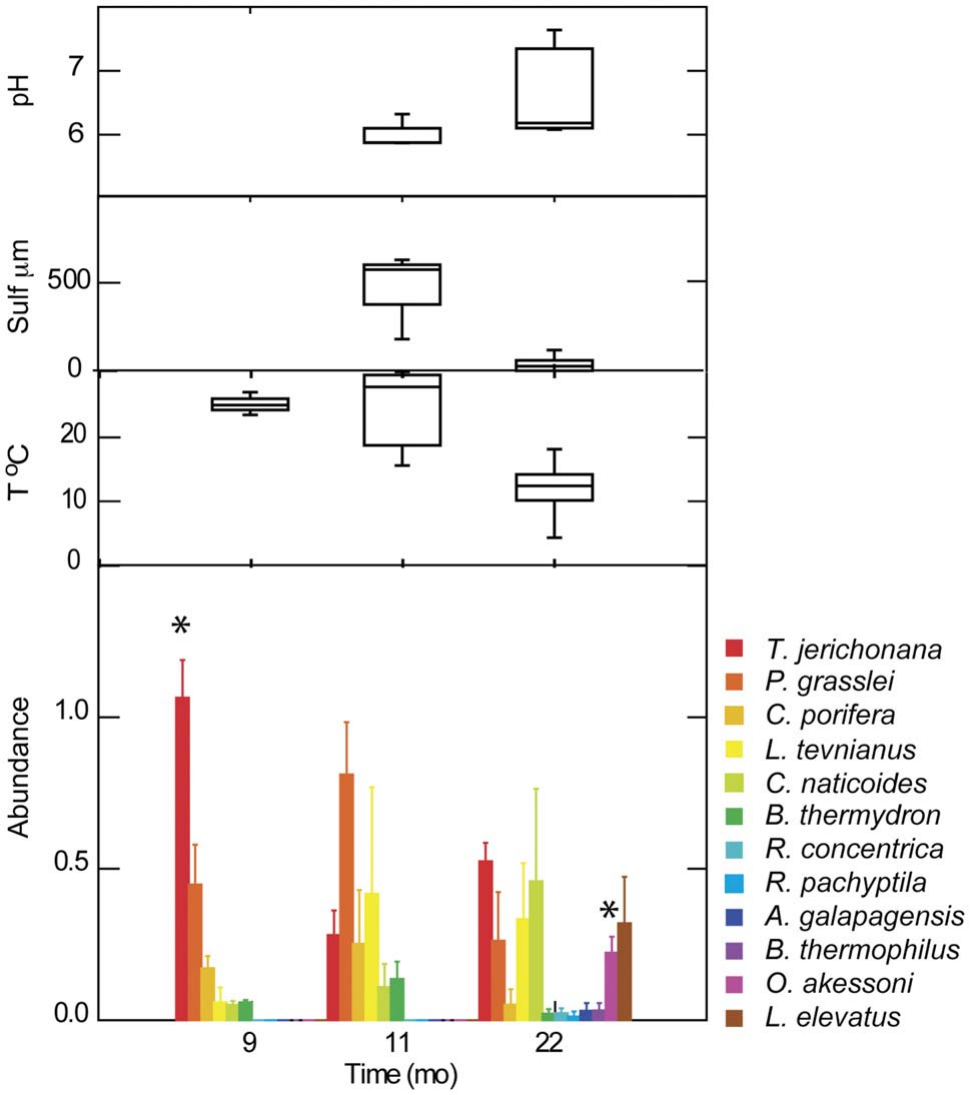
Temporal change in fauna and fluid environment at P-vent. Species abundance (mean and standard error; arcsine square-root transform of relative abundance) of selected species colonizing at times 9, 11 and 22 months after the eruption in hot habitat. Asterisk denotes abundance significantly greater than at least one other time (Table S3). Environmental conditions, displayed as box plots (median, quartiles, range) measured in vicinity of colonization surfaces with Alvin T-probe alone (9 months) or in combination with electrochemical sensors coupled with T-probes (11 and 23 months).

The dominant initial pioneer species recovered 9 months after the eruption in hot habitat at P-vent were siboglinid tubeworms (identified tentatively as *T. jerichonana*) and the polychaete *Paralvinella grasslei* (Fig. 4). Other notable pioneers included the gastropods *Ctenopelta porifera*, *Lepetodrilus tevnianus*, and *Cyathermia naticoides*. The crab *Bythograea thermydron* was prominent in colonization samples; an observation consistent with an increase in post-eruption larval abundance and reports of unusually high abundances of juvenile crabs in this area directly after the eruption (authors’ unpublished data). Pioneers *C. porifera* and *L. tevnianus* had not been found in previous colonization studies in the 9°50′N region [7], [15]; the nearest adult *C. porifera* had been reported from 13°N. Pioneers in the 9-month samples at Ty/Io, which was a hot habitat at the start of the deployment, differed in that *L. tevnianus* and the polychaetes *Amphisamytha galapagensis* and *Ophryotrocha akessoni* were numerically dominant, and the mussel *B. thermophilus,* which is typically considered a late-succession species, was present (Table 3). These differences in composition were probably due to a marked decrease in habitat temperature over the course of the deployment (temperatures dropped to near ambient). Measurements were not continued at Ty/Io, and those data were not included in further analyses.

Over the next year at P-vent (11 and 22 months post-eruption) pioneer species persisted, but a suite of new species arrived including the prominent gastropod *Lepetodrilus elevatus* and polychaetes *A. galapagensis* and *O. akessoni.* By 22 months, siboglinid tubeworms were large enough to identify, revealing *Tevnia jerichonana* as dominant, and a few individuals of *Riftia pachyptila* (Table 3). Some of the non-*Riftia* specimens were still too small to differentiate conclusively between *T. jerichonana* and *Oasisia alvinae*, but as no identifiable *O. alvinae* were found in any samples or observed in the surrounding community, these uncertain specimens were all assigned to *T. jerichonana*.

Several of the initial pioneer species at P-vent decreased in mean relative abundance between the 9-and 22-month recoveries (Fig. 4); this decrease was significant for *T. jerichonana* (ANOVA F = 32.7, df = 2,6, P = 0.001; Table S3), and notable (but not significant) for *C. porifera*. In contrast, the pioneer *L. tevnianus* increased in relative abundance, although not significantly. The general decrease in relative abundance of pioneers was due in part to the arrival of *O. akessoni*, *L. elevatus*, and numerous rare species (Table 3). In some cases (e.g., *R. concentrica, L. elevatus*), a marked increase in abundance in the 22-month recovery may have been obscured in the statistical analysis by high variation and the low number of replicates. Of the later arrivals, only *O. akessoni* was significantly more abundant at 22 months when corrected for multiple tests (F = 30.7, df = 2,6, P = 0.001; Table S3).

**Figure 5:**
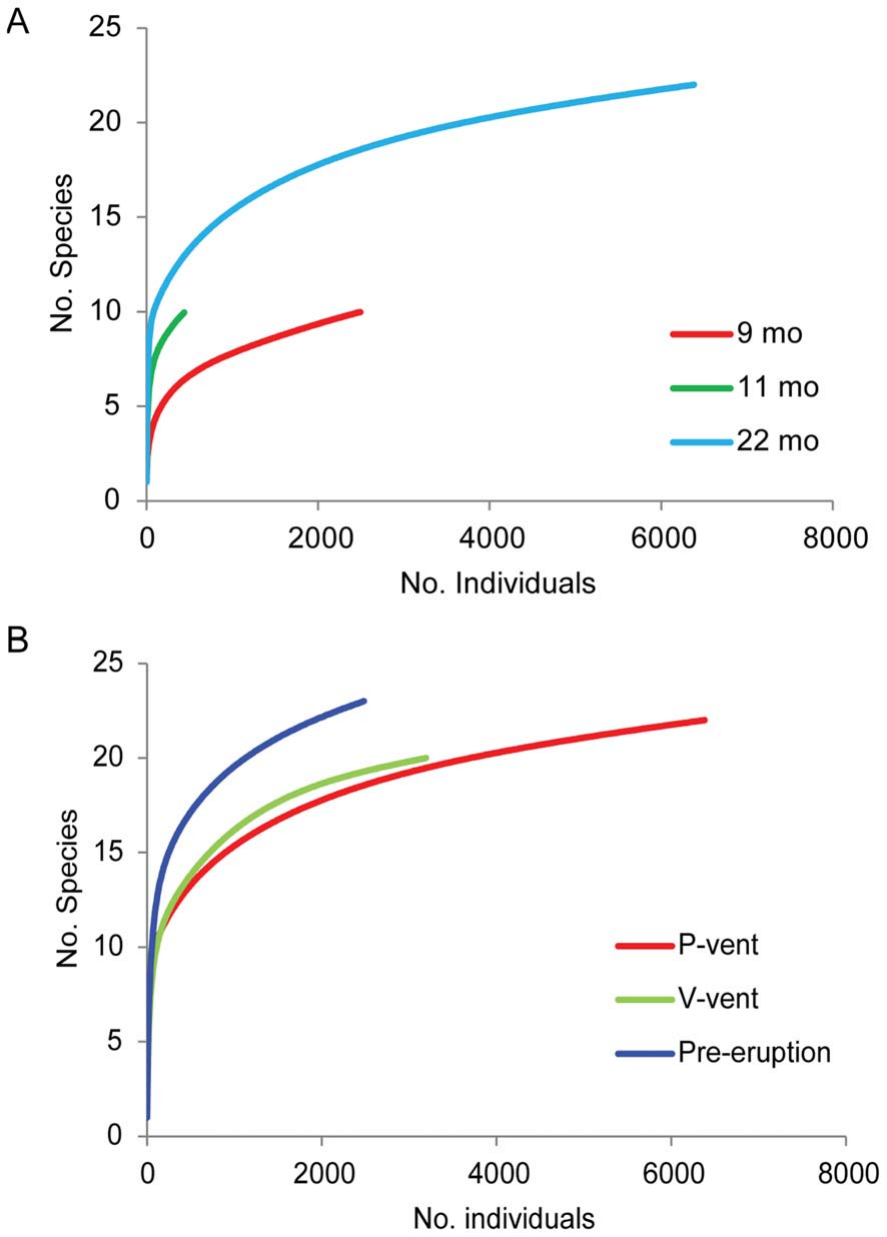
Species diversity as a function of number of individuals from rarefaction analysis. Curves are pooled over three replicates for each recovery date (9, 11 and 22 months after the eruption) in the analysis of Time (A) and for each disturbance history (disturbed at P-vent, undisturbed at V-vent and undisturbed Pre-eruption) in analysis of Disturbance (B).

The appearance of rare species at P-vent at 22 months raised the total number of species from 10 to 22. A rarefaction analysis indicates that this is a real increase in species diversity (Fig. 5A) and not simply a result of higher numbers of individuals, although none of the sample sets approached the numbers needed to characterize the full diversity.

Over the course of the study, the seawater temperature at colonization surfaces in the hot habitat at P-vent decreased substantially (mean from 17° to 9°C, maximum from 30° to 14°C). This decrease suggests a lower contribution of the vent fluid to habitat at 23 months, reflecting an increased dilution with seawater. This habitat was still considered hot and remained inhabited by siboglinid tubeworms, but was nearing a transition to warm. The median pH was stable (6.3 to 6.4) from 11 to 22 months (Table 2), while the minimum pH increased (5.8 to 6.1). Free sulfide decreased substantially over the same period (median from 210 to 16 µM; maximum from 765 to 115 µM). These changes in habitat conditions are consistent with the combination of both an increased dilution of the fluid and change in fluid properties as described in the previous section.

### Effect of Disturbance

**Figure 6:**
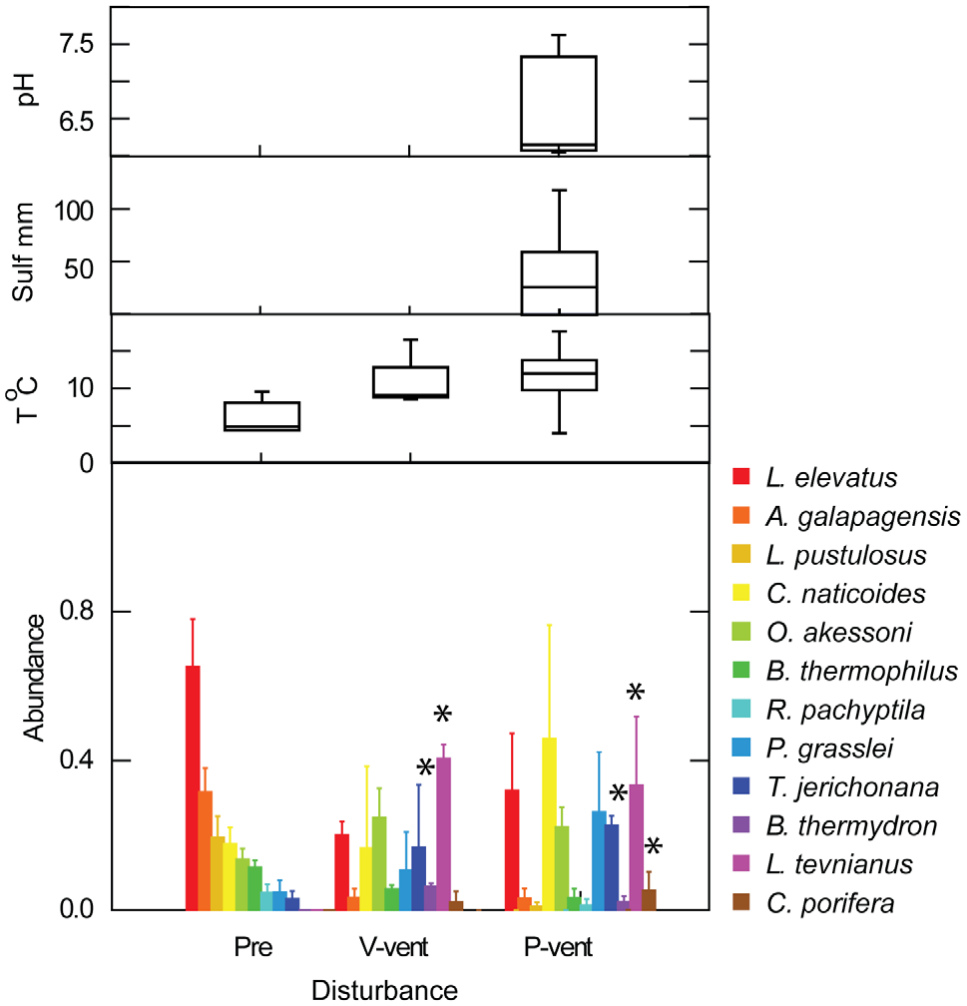
Variation of fauna and fluid environment with disturbance history at P-vent, V-vent and pre-eruption. Species abundance (mean and standard error; arcsine square-root transform of relative abundance) of selected species in pre-eruption samples in 9°50′N region (Pre) and in 22-month, post-eruption samples at P- and V-vents, all in hot habitat. Asterisk denotes abundance significantly greater than at least one other condition in ANOVA (Table S3) or Kruskal Wallace test. Environmental conditions, displayed as box plots (median, quartiles, range) measured in vicinity of colonization surfaces with Alvin T-probe alone (Pre-eruption and V-vent) or in combination with electrochemical sensors coupled with T-probes (P-vent).

Twenty-two months after the eruption, species composition at P-vent remained distinctly different from pre-eruption faunas at Biovent and East Wall in similar habitat (Fig. 3B), indicating that P-vent had not returned to a pre-eruption state. Of the 30 species found in hot habitat before the eruption, only 15 had reestablished at P-vent hot habitat by 22 months afterward (Table 3), although four others (not including pooled groups of unknown polychaetes and unknown gastropods) had been observed in post-eruption warm habitat at P-vent (11 months) or at Ty/Io (9 months). Six new species were resident, including pioneers *C. porifera*, *L. tevnianus* and *B. thermydron* (Fig. 6), and later (rare) arrivals *Branchinotogluma* sp., *Branchiplicatus cupreus* and *Prionospio sandersi* (Table 3). The group *Lepetodrilus* spp. was not included in this count because it probably comprised mostly *L*. *tevnianus*. It is important to point out that the crab *B. thermydron*, although not found in the pre-eruption colonization samples used herein, was well established in the 9°50′N community before the eruption [21]. The V-vent community at 22 months was distinct from pre-eruption faunas, suggesting that the eruption had an influence there even though no lava covered the site and no eruption effect was detected in post-eruption video images (authors’ unpublished data). Communities at P-vent and V-vent were, in general, more similar to each other in species composition than to those in pre-eruption sites (Fig. 3B). Rarefaction analysis showed that P-vent and V-vent communities were similar in diversity, and less diverse than pre-eruption communities (Fig. 5B).

A significant increase in abundance between P-vent and pre-eruption communities was detected for *B. thermydron* (ANOVA; F = 9.6, df = 2,10, P = 0.005; Table S3), but not for *C. porifera* or *L. tevnianus*. Heteroscedacity (unequal variance) was apparent for all three species, even after data transformations. An alternative, non-parametric, test (Kruskal Wallace; Systat v. 11), which is less sensitive to variance equality, detected significant (P<0.05) increases between P-vent and pre-eruption sites for all three species. Pioneers *C. naticoides, P. grasslei,* and *T. jerichonana* persisted at P-vent at 22 months (Fig. 6), but were not significantly more abundant than in pre-eruption sites. Surprisingly, several pioneers had also become established at V-vent even though the existing community had remained intact there after the eruption. Pioneers *B. thermydron* and *L. tevnianus* were significantly more abundant at V-vent than in pre-eruption sites (ANOVA; Fig. 6, Table S3).

The dominant two species in pre-eruption communities, *L. elevatus* and *A. galapagensis*, had notably lower relative abundance at post-eruption P- and V-vents, but the differences were not significant when corrected for multiple tests. Most of the species that were present in pre-eruption sites, but not at P-vent, were rare in hot habitat (Table 3).

Temperature ranges experienced on experimental surfaces at P-vent and V-vent at 22 months were similar (Fig. 6), but higher than those recorded at pre-eruption surfaces. The habitat in all cases was hot, and supported tubeworms, but environmental conditions may have played a role in the presence/absence of some species in the pre-eruption sites (see *Influence of habitat*). Free sulfide and pH were not measured at pre-eruption sites or at 22 months post-eruption at V-vent, but measurements from V-vent and P-vent at 11 months showed that the relationship between free sulfide and temperature in hot (tubeworm) habitat was similar between the two sites (Fig. 2). For a given temperature, pH was slightly less acidic at V-vent, at least in the hottest part of the habitat.

### Influence of Habitat

**Figure 7:**
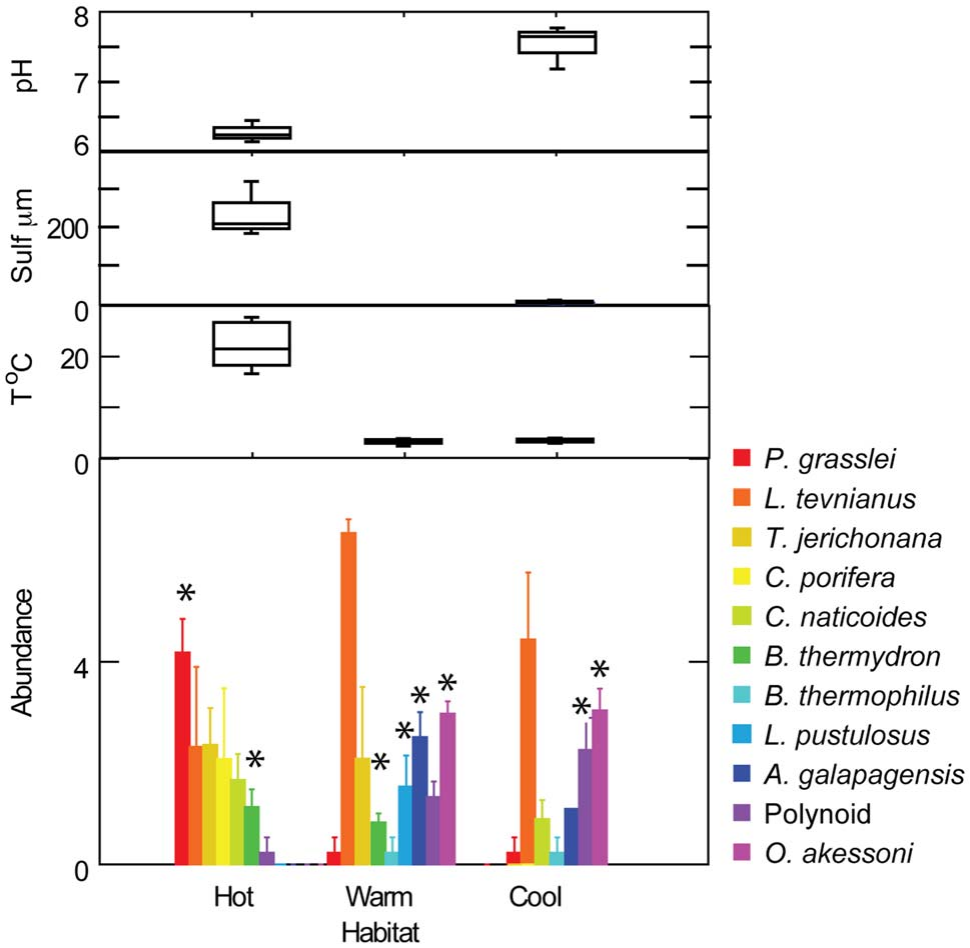
Variation of fauna and fluid environment with habitat at P-vent. Abundance (mean and standard error; log transformed) of selected species in hot, warm and cool habitat at 11 months. Asterisk denotes abundance significantly greater than one or both other conditions (Table S3). Environmental conditions are displayed as box plots (median, quartiles, range) measured in vicinity of colonization surfaces with Alvin T-probe and electrochemical sensors coupled with T-probes.

Species composition of colonists in P-vent differed between hot, warm and cool habitats at 11 months post-eruption, with the exception of one cool sample that clustered with warm communities (Fig. 3C). Pioneer species tended to be most abundant in hot habitat. This pattern was significant for the polychaete *P. grasslei* (F = 43.4, df = 2,6, P<0.001; Table S3) and the crab *B. thermydron* (F = 11.7, P = 0.009; Table S3) (Fig. 7). Small tubeworms (probably *T*. *jerichonana*) were absent, as expected, from the cool habitat, and *C. porifera* was absent from both warm and cool habitat. The gastropod *L. tevnianus* differed from other pioneers in that it occurred in all habitats and was generally more abundant in warm than hot habitat (F = 5.4, df = 2,6, P = 0.045, not significant when corrected for multiple tests; Table S3). Later-arriving species tended to be more abundant in cool and/or warm habitat than hot. This pattern was significant for *A. galapagensis* (F = 5.0, P = 0.003), *O. akessoni (*F = 64.8, P = <0.001), *L pustulosus* (F = 10.8, P = 0.010), and polynoids (F = 11.8, P = 0.008).

In the vicinity of P-vent colonization experiments at 11 months, median temperatures ranged from almost 17°C in hot habitat to near-ambient (2°C) in cool (Table 2). Median pH increased from 6.3 to 7.7, and median free sulfide decreased from 210 µM to 1 µM. All physical and chemical characteristics were more variable in hot than cool habitat, reflecting high variation in vent fluid-seawater mixing ratio over small spatial and temporal scales.

Community composition of pioneers (9 months) at the Ty/Io site was dominated by *L. tevnianus,* making it resemble later stages at P-vent (11 and 22 months) (Table 3). This comparison is complicated because both habitat and site differed, but it demonstrates that at least some later-arriving species at P-vent (e.g., the mussel *B. thermophilus*, gastropods *Clypeosectus delectus* and ‘pointy apex’, and polychaetes *A. galapagensis* and *O. akessoni*) were available in the region as larvae as early as 5 to 9 months after the eruption (Table 3). Their absence in the P-vent hot habitat at 9 months is likely due to the environmental conditions, which were at the high end of the temperature range.

## Discussion

Nearly two years after the 2006 eruption, the community colonizing the disturbed P-vent site included many species found at established pre-eruption sites, but remained distinctly different, due to continued presence of new pioneer species *Lepetodrilus tevnianus* and *Ctenopelta porifera*, and absence of numerous rare species. Surprisingly, the community at V-vent, which was not covered by lava during the eruption, was more similar to disturbed than to pre-eruption communities. One reason for this similarity was the post-eruption invasion of V-vent by *L. tevnianus*. In both cases, the question remains whether these patterns are due to post-eruption changes in environmental conditions (vent fluid composition and flux), or to larval supply. These alternative scenarios fit into the context of metacommunity theory [25] as examples of ‘species sorting’ where species’ responses to local abiotic environment in patches (e.g., [26]) influence regional patterns, and ‘mass effects’ where patches with high reproductive output influence the dynamics of neighboring patches through dispersal [27].

For some prominent species, post-eruption changes in abundance were likely due to changes in habitat – i.e., the decrease in the vent fluid supply to communities (and corresponding decrease in temperature and free sulfide and increase in pH) and in the sulfide to temperature ratio of the fluid. For instance, *O. akessoni*, which was associated with warm and cool habitat, increased in relative abundance over time, while *C. porifera* and *B. thermydron*, which were associated with hot habitat, decreased. Similarly, for some species, the difference between post-eruption (22 months at P-vent) and pre-eruption abundance may have reflected the slightly lower temperatures (and, presumably, lower vent fluid contribution in habitats) in pre-eruption sites. *A. galapagensis*, which was associated with warm habitat, was less abundant in post-eruption hot habitat, while *B. thermydron* was more abundant. This difference in *B. thermydron* was consistent with a post-eruption increase in their larval abundance [9] and in visual observations of juveniles, but may also have been influenced by a change from blocks to sandwiches, which provide refuge for the crabs during recovery.

For many species, however, differences in abundance over time and across disturbance conditions were not explained by response to environmental conditions. The post-eruption arrival of *C. porifera* and *L. tevnianus* was due to a combination of their availability in the plankton immediately after the eruption and their ability to settle into the unoccupied habitat. Their absence before that time is not due solely to lack of suitable environment. *C. porifera* appears to inhabit the high end of the temperature range of hot habitat. Although chemical conditions might have been slightly different, such hot environments had been observed near black smokers before the eruption. There were no established adults at nascent vents after the eruption, so larvae arriving from elsewhere faced no competition from the benthos, and possibly reduced competition from other larvae, since local sources were eliminated. This window of opportunity for immigrants was not a mass effect per se, because larval abundances weren’t necessarily high, but rather a consequence of reduced competition and predation from locals.

*L. tevnianus* did not decrease in relative abundance over time at P-vent, despite the decrease in vent fluid flux at that site, and appears to have broad environmental tolerances. Furthermore, *L. tevnianus* colonized V-vent, where it had not been observed before the eruption. We suspect that it was able to invade this densely populated community only after it became dominant at P-vent and other nascent sites and started to dominate local larval supply. This type of local mass effect is consistent with observations of high temporal [28], [29] and spatial [30] variation in larval availability at vents along segments of mid-ocean ridge.

Another prominent species, *L. elevatus*, did not appear to colonize in response to changing environmental conditions. Past observations show that this species is more abundant in hot habitat than warm or cool [18], but it did not decrease over time at P-vent. Instead, it arrived in high numbers only after 22 months. This delayed arrival differs from the 1991 eruption when *L. elevatus* was very abundant (100*–*500 ind. m^−2^) within 11 months of the event [3]. It is possible that *L. elevatus* can only inhabit hot habitat when the sulfide-to-temperature ratio is low, or it may be a late-stage colonist due to life-history characteristics that resulted in low larval supply in early post-eruption months. We think that the most likely explanation for its delayed arrival after the 2006 eruption is that its congener *L. tevnianus* colonized first and impeded its ability to settle successfully. At this point it is not clear whether the pioneer *L. tevnianus* will persist as the dominant gastropod, perhaps leading to an alternative stable state in the grazers, or whether *L. elevatus* will take over in the long term.

After two years, the fauna at the eruption-disturbed site (P-vent) was still missing many rare species that were present in similar habitat before the eruption. Biological factors, such as negative interactions with pioneer colonists [12], or poor dispersal capabilities [11], may have contributed. Environmental conditions need also be considered, but chemical measurements indicate that post-eruption conditions in hot habitat 22 months after the eruption did not differ substantially from those in pre-eruption tubeworm habitats. The free sulfide to temperature ratio (3 µM °C^−1^) fell within the range of most tubeworm-dominated pre-eruption habitats (<1 to 11 µM °C^−1^) [24], [31], and the pH - T trend was also very similar. One notable difference, however, between pre- and post-eruption hot habitat was the prominence of thickets of large tubeworms (*R. pachyptila*) before the eruption, in contrast to the patches of the smaller tubeworm (*T. jerichonana*) that characterized the post-eruption hot habitat after 22 months. Tubeworms are considered foundation species [32], and may influence other colonists, including microbes, in subtle ways not reflected in fluid chemistry or temperature. In particular, the longer *R. pachyptila* tubes may provide surfaces for colonization across a broader temperature and chemistry gradient.

An intriguing characteristic of successional change at P-vent was the change over time of trophic groups. The community was initially (at 9 months) dominated by autotrophic siboglinid tubeworm symbioses and mat-forming microbes. At 11 months, gastropod grazers had become more important, and by 22 months, a diverse group of detritovores (polychaetes, crustaceans) had become established. This sequence may have been a consequence of increasing availability of free-living benthic microbial prey (including eukaryotes) and particulate organic carbon over time.

The community at V-vent, which was at the margin of the eruption but not impacted by lava, was clearly influenced by the arrival of post-eruption species (*L. tevnianus*, *B. thermydron*, *C. porifera*), causing it to diverge from the pre-eruption type of community and become similar to that at P-vent. Physical and chemical conditions at P-vent and V-vent were quite similar at 11 months after the eruption, with a sulfide to temperature anomaly ratio around 15 µM °C^−1^ at both sites (Fig. 2), although the amplitude of variation in temperature and free sulfide was higher at P-vent. The range of temperature at V-vent was similar to that observed in hot habitat in transition from *R. pachyptila* to mussel pre-eruption at Biovent in 2002 with a slightly lower sulfide to temperature ratio (11 µM °C^−1^ [24]). We assume it is characteristic of V-vent before the eruption (unfortunately we do not have pre-eruption habitat measurements from this site). We suspect that community composition at V-vent was influenced by species that had first gotten established at the disturbed sites and then invaded undisturbed ones. While it is also possible that these species colonized V-vent directly, their larval supply was low and it is difficult to explain how they could infiltrate an undisturbed site when they had not done so prior to the eruption. If the vent fluid flux at V-vent did increase after the eruption, that might explain, at least in part, why *C. porifera,* which inhabits the high end of temperature range, could colonize. The invasion by *L. tevnianus*, however, and its absence at pre-eruption sites, probably is not due to environmental differences, because this species has a broad thermal distribution and occurs in high abundance in warm habitat.

Some of the species monitored in our colonization samples can be identified to species in camera surveys (e.g., siboglinid tubeworms, mussels, bythograeid crabs), making it possible to compare succession of select species between the 1991 and 2006 eruptions. The ‘*Tevnia-Riftia-*mussel’ transition was well documented after the 1991 event by Shank et al. [3]. At 11 months after the 1991 eruption, the authors observed *T. jerichonana* as the dominant tubeworm. By 32 months, the larger tubeworm *R. pachyptila* had largely displaced it, and by 42 months small individuals of the mussel *B. thermophilus* were visible. In our study, *R. pachyptila* had not colonized by 11 months, and remained rare at 22 months, while *T. jerichonana* and associated fauna (e.g. *L. tevnianus*) continued to dominate. Visual seafloor observations in October 2008 (32 months post-eruption, authors’ unpublished data) showed that *T. jerichonana* remained dominant in the region, although thickets of *R. pachyptila* were developing. The mussel was observed at 9, 11 and 22 months post-eruption, but in very low numbers. These observations suggest that the *Tevnia-Riftia* transition may have been slower to develop after the 2006 event.

After both eruptions, environmental conditions changed over the years following the event. Vent fluid flux decreased over a 55-month period after the 1991 eruption, and a similar decrease was observed after 2006. In the earlier eruption, the sulfide to temperature ratio remained higher in vent fluids fuelling tubeworm habitats at 32 and 42 month post-eruption (respectively 30–39 and 22–33 µM °C^−1^, calculated from data in Shank et al. [3]), than at 11 months after the 2006 eruption (15 µM °C^−1^ at P-vent and V-vent). Only at 55 months after the 1991 eruption did environmental conditions (sulfide to temperature ratio of 6 to 29 µM °C^−1^) become similar to those we observed. Our chemical measurements reflect conditions during early settlement and may not be strictly comparable with the properties of vent fluids fuelling mature biological assemblages (i.e. tubeworm bushes or mussel beds), as described in Shank et al. [3]. Furthermore, total sulfide concentration could include a significant fraction of iron sulfide in these earlier studies, while we only measured the available free sulfide fraction. Nevertheless, the delay in succession after the 2006 event did not correspond to a concomitant delay in vent fluid chemistry changes.

In summary, the trajectory of succession after the 2006 eruption differed both qualitatively and dynamically from that described after 1991. Two new species arrived, one of which (*L. tevnianus*) persisted as a dominant, delaying and possibly outcompeting congener *L. elevatus*. The tubeworm *T. jerichonana* continued to dominate two years after the eruption, and *R. pachyptila* had not yet taken over even after nearly three years. Over the two-year period following the eruption, the temperature of vent fluid habitat decreased, with associated decrease in free sulfide and increase in pH. The sulfide to temperature ratio also changed over time, decreasing back to the pre-eruption values reported in Le Bris et al. [24] and Nees et al. [31]. However, instead of facilitating the transition to faster growing *R. pachyptila* and the pervasive mussel *B. thermophilus*, the decrease in flow rate and lower energy of the fluid (as estimated from sulfide to temperature ratios) allowed the persistence of pioneers. Additional species arrived over time, but diversity remained lower than pre-eruption levels. In this early period of succession, larval availability and species interactions appear to have had a strong influence on community structure, although environmental conditions clearly influenced the abundance of some species.

## Supporting Information

Figure S1 **Temperature records from different habitats at P-vent and V-vent.** Habitats were characterized as hot (red), warm (green), or cool (blue). Measurements recorded every 30 min by Hobo temperature logger. Each logger was placed near a cluster of colonization surfaces. Loggers at P-vent were recovered during the Dec. 2006 cruise and re-deployed in the same site and habitat.
(TIF)

Table S1 **Chemical measurements analyzed by scan.**
(PDF)

Table S2 **Abundance of species (taxa) on individual colonization sandwiches and blocks.** Sandwiches were recovered from hot, warm or cool habitat after the eruption (9, 11 or 22 months) at P-vent, Ty/Io, and V-vent. Blocks were recovered from hot habitat before the eruption (Pre) at Biovent and Worm Hole. Temperature (maximum on recovery) measured at surfaces with Alvin probe.
(PDF)

Table S3 **Analysis of variance (ANOVA) for Time.** Species abundance was compared between colonization surfaces recovered at 9, 11 and 22 months after eruption from the hot environment at P-vent (SS = sum of squares, df = degrees of freedom, MS = mean squares). Data transformed as arcsine(square-root(relative abundance)). Post-hoc Tukey test used when P<0.05. Significant differences (bold) include Bonferroni correction for multiple tests, with significance level adjusted as appropriate for pioneer colonists (6 species, P<0.008) and later arrivals (6 species, P<0.008). *T. jerichonana* group includes small vestimentiferan tubeworms.
(PDF)

Table S4 **Analysis of variance (ANOVA) for Disturbance.** *ANOVA compromised by heteroscedasticity; Kruskal Wallis test significant P<0.005. Species abundance was compared between colonization surfaces recovered at sites with different disturbance histories: P-vent (post-eruption, disturbed), V-vent (post-eruption, undisturbed), and Pre-eruption. Data are transformed as arcsin(square-root(relative abundance)). Post-hoc Tukey test used when P<0.05. Significant differences (bold) include Bonferroni correction for multiple tests, with significance level adjusted as appropriate for pioneer colonists (6 species, P<0.008) and later arrivals (6 species, P<0.008).
(PDF)

Table S5 **Analysis of variance (ANOVA) for Environment at 11 months.** *Probably *Tevnia jerichonana.* Species abundance was compared across different thermal environments (hot, warm, cool) at P-vent, 11 mo after eruption. Data are transformed ln(abundance)+1). Post-hoc Tukey test used when P<0.05. Significant differences (bold) include Bonferroni correction for multiple tests, with significance level adjusted for pioneer colonists (6 species, P<0.008) and later arrivals (5 species, P<0.01).
(PDF)

Table S6 **Analysis of variance (ANOVA) for Environment at 9 months.** *Probably *Tevnia jerichonana.* Species abundance was compared between colonization surfaces recovered from different thermal environments (hot at P-vent and warm at Ty/Io), 9 mo after eruption. Data are transformed ln(abundance+1). Post-hoc Tukey test used when P<0.05. Significant differences (bold) include Bonferroni correction for multiple tests, with significance level adjusted as appropriate for pioneer colonists (6 species, P<0.008) and later arrivals (5 species, P<0.01).
(PDF)
