# Supplementary figures and images for: Detecting the Influence of Initial Pioneers on Succession at Deep-Sea Vents
Source: PLoS One. 2012 Dec 4;7(12):e50015. doi: 10.1371/journal.pone.0050015 (PMC3514232; doi:10.1371/journal.pone.0050015)

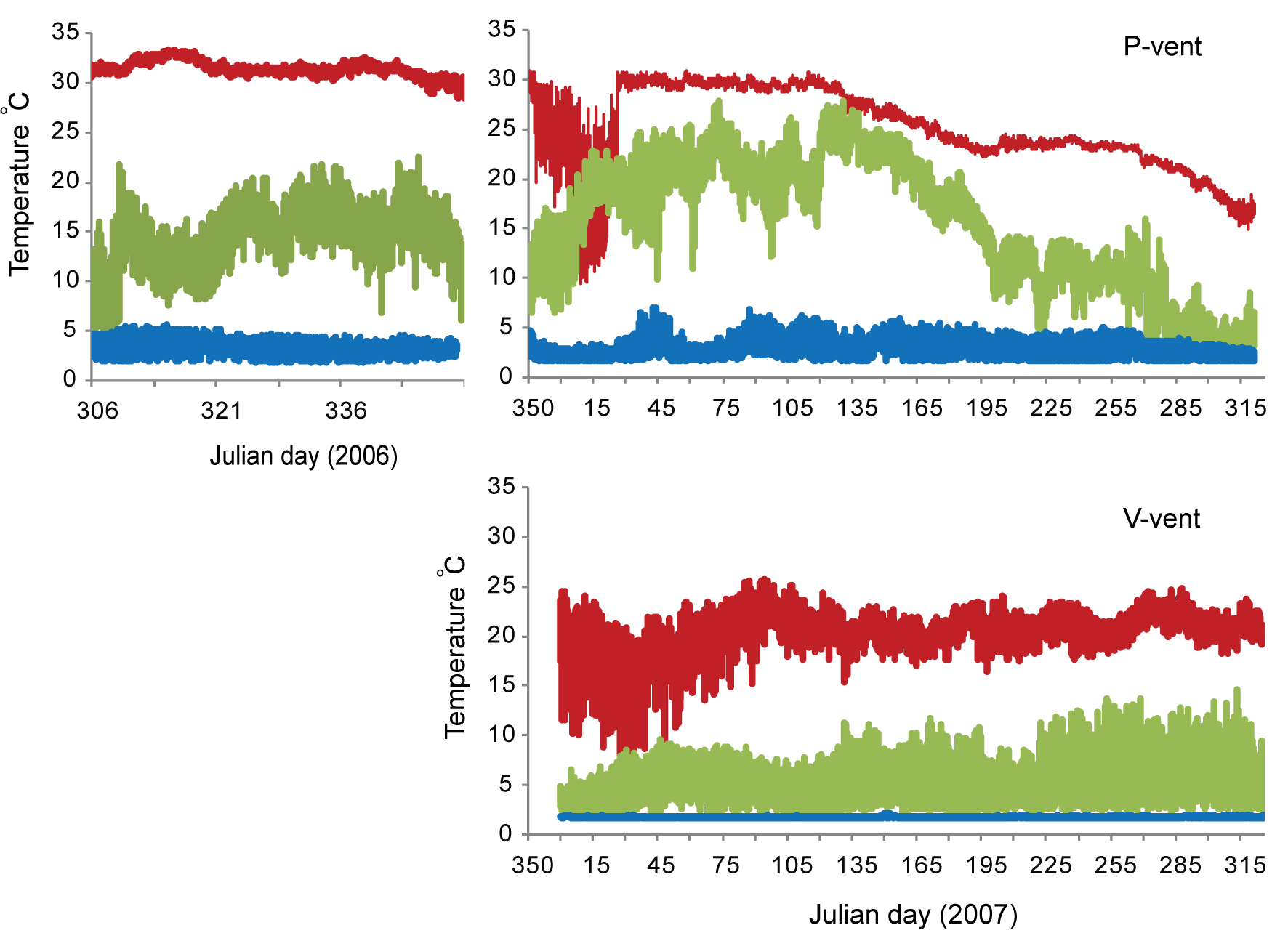

Supplement: Figure S1 — Temperature records from different habitats at P-vent and V-vent. Habitats were characterized as hot (red), warm (green), or cool (blue). Measurements recorded every 30 min by Hobo temperature logger. Each logger was placed near a cluster of colonization surfaces. Loggers at P-vent were recovered during the Dec. 2006 cruise and re-deployed in the same site and habitat. (TIF) [file pone.0050015.s001.tif]
