# Supplementary material for: Detecting the Influence of Initial Pioneers on Succession at Deep-Sea Vents
Source: PLoS One. 2012 Dec 4;7(12):e50015. doi: 10.1371/journal.pone.0050015 (PMC3514232; doi:10.1371/journal.pone.0050015)
Supplement: Table S1 — Chemical measurements analyzed by scan. (PDF) [file pone.0050015.s002.pdf]

Dive	Scan	Cruise	Site	Exp	Temperature °C				pH				H_2S µM			
					Med	Max	Mean	St Dev	Med	Min	Mean	St Dev	Med	Max	Mean	St Dev
4288	1	Dec. 2006	P-Vent	Cool	3.3	3.9	3.4	0.2	7.4	7.2	7.4	0.1	3	9	3	2
	2				3.2	4.1	3.5	0.3	7.8	7.6	7.8	0.1	2	4	2	1
	3				2.5	3.1	2.6	0.2	8.0	7.8	7.9	0.1	0	1	0	0
	4		P-Vent	Hot	28.4	29.3	28.6	0.4	5.9	5.9	5.9	0.04	474	619	473	54
	5				12.4	15.4	13.5	1.4	6.5	6.3	6.5	0.2	91	176	96	43
	6				27.5	29.8	28.3	1.1	6.0	5.9	5.9	0.04	411	564	430	60
4292	25	Dec. 2006	P-Vent	Hot	10.8	16.8	11.7	2.7	6.7	6.5	6.7	0.2	103	183	104	45
	26				15.3	22.0	17.0	3.7	6.3	6.1	6.4	0.2	158	319	170	97
	27				14.9	21.5	16.1	2.2	6.4	6.2	6.4	0.1	140	209	141	31
4293	1	Dec. 2006	V-Vent	Hot	13.2	17.3	13.9	1.9	6.8	6.7	6.9	0.1	197	462	216	104
	2				5.4	8.8	6.2	1.2	7.3	6.9	7.3	0.2	50	184	54	36
	3				15.9	19.8	17.1	1.9	6.8	6.6	6.8	0.2	209	300	189	79
	4				10.3	16.0	11.2	1.6	6.9	6.7	7.0	0.2	121	248	124	61
	5				4.3	7.0	4.8	0.9	7.4	7.2	7.4	0.1	20	50	23	11
	6				7.5	9.5	8.2	1.0	7.1	6.9	7.2	0.2	76	149	75	43
4293	7	Dec. 2006	V-vent	Warm	2.6	3.4	2.7	0.2	7.8	7.7	7.8	0.03	3	5	3	1
	8				2.7	3.4	2.9	0.3	7.8	7.7	7.8	0.04	4	8	4	2
	9				2.7	3.5	2.9	0.3	7.8	7.7	7.8	0.05	4	9	5	2
	10				2.5	3.4	2.8	0.3	7.8	7.6	7.7	0.1	0	1	0	0
	11				6.3	7.5	6.7	0.8	7.3	7.2	7.3	0.1	16	36	18	10
	12				2.8	3.8	3.1	0.4	7.7	7.5	7.7	0.1	1	5	2	1
4293	13	Dec. 2006	V-vent	Cool	1.9	2.1	1.9	1.9	7.9	7.9	7.9	0.00	0	0	0	0
	14				1.9	2.1	1.9	1.9	7.9	7.9	7.9	0.01	0	0	0	0
4394	7	Jan. 2008	P-Vent	Hot	2.4	2.9	2.5	0.2	7.7	7.6	7.7	0.0	0	0	0	0
	8				2.5	4.2	3.0	0.6	7.6	7.3	7.6	0.1	0	0	0	0
	9				11.1	13.8	12.2	1.3	6.2	6.1	6.3	0.1	1	21	8	9
4398	6	Jan. 2008	P-Vent	Hot	12.0	14.0	12.7	0.9	6.3	6.2	6.3	0.2	25	30	24	5
	7				9.1	11.9	9.9	1.2	6.4	6.2	6.4	0.2	27	58	27	17
	8				6.4	12.2	7.9	2.1	6.2	6.1	6.3	0.2	72	115	63	35
