# Supplementary material for: Detecting the Influence of Initial Pioneers on Succession at Deep-Sea Vents
Source: PLoS One. 2012 Dec 4;7(12):e50015. doi: 10.1371/journal.pone.0050015 (PMC3514232; doi:10.1371/journal.pone.0050015)
Supplement: Table S2 — Abundance of species (taxa) on individual colonization sandwiches and blocks. Sandwiches were recovered from hot, warm or cool habitat after the eruption (9, 11 or 22 months) at P-vent, Ty/Io, and V-vent. Blocks were recovered from hot habitat before the eruption (Pre) at Biovent and Worm Hole. Temperature (maximum on recovery) measured at surfaces with Alvin probe. (PDF) [file pone.0050015.s003.pdf]

Treatment	9 mo, P vent, Hot			9 mo, Ty/Io, Warm			11 mo, P vent, Hot			11 mo, P vent, Warm			11 mo, P vent, Cool			22 mo, P vent, Hot			22 mo, V vent, Hot			Pre, Biovent, Hot				Pre, WH, Hot	
**Temp (°C)**	26.7	23.2	24.7	2.2	2.2	6.5	18.5	27	28	9.6	7	9	3.3	3.3	3.7	10	17.9	14.7	9.3	16.8	8.8	4.6	5.5	9.8	4.6	7.3	4.7
**Taxon**																											
Bathymargarites symplector	0	0	0	0	0	0	0	0	0	0	0	0	0	0	0	0	0	0	0	0	0	0	0	0	0	0	1
Clypeosectus delectus	0	0	0	4	0	0	0	0	0	0	0	0	3	6	1	0	0	0	0	0	0	0	0	0	0	0	1
Ctenopelta porifera	20	10	45	0	0	1	0	31	15	0	0	0	0	0	0	0	2	14	0	3	0	0	0	0	0	0	0
Cyathermia naticoides	1	1	6	0	0	1	0	6	3	0	0	0	2	0	1	0	973	390	121	0	0	5	9	139	27	25	2
Eulepetopsis vitrea	0	0	0	2	0	0	0	0	0	0	0	0	0	0	1	0	0	0	0	0	0	0	0	0	0	0	0
Gorgoleptis spiralis	0	0	0	2	1	0	0	0	0	0	0	0	1	4	3	0	0	0	0	0	0	3	0	0	0	0	0
Gorgoleptis emarginatus	0	0	0	0	0	0	0	0	0	1	0	2	0	0	0	0	0	0	0	0	0	0	0	0	0	0	0
Lepetodrilus cristatus	0	0	0	0	0	0	0	0	0	0	0	0	0	0	0	0	0	0	0	0	0	14	2	0	11	0	0
Lepetodrilus elevatus	0	0	0	0	0	2	0	0	0	2	0	0	0	0	0	287	422	7	24	41	9	60	42	479	254	1949	683
Lepetodrilus ovalis	0	0	0	0	0	0	0	0	0	0	1	2	0	0	0	1	0	0	0	0	1	1	0	0	0	1	1
Lepetodrilus pustulosus	0	0	0	0	0	0	0	0	0	9	4	1	0	0	0	1	0	0	0	0	0	1	10	13	32	45	48
Lepetodrilus tevnianus	1	0	21	378	1268	1557	52	0	19	466	928	769	18	56	566	433	278	8	62	125	73	0	0	0	0	0	0
Lepetodrilus spp.	0	0	1	1738	633	1056	0	0	0	282	428	291	268	465	527	196	101	1	17	0	13	0	0	0	0	0	0
Pachydermia laevis	0	0	0	2	0	0	0	0	0	0	0	0	0	0	0	0	0	0	0	0	0	0	0	0	0	0	0
Planorbidella planispira	0	0	0	1	0	0	0	0	0	0	0	0	0	0	0	0	0	0	0	0	0	0	0	0	0	0	0
Rhynchopelta concentrica	0	0	0	0	1	0	0	0	0	0	0	0	0	0	0	2	3	0	0	0	0	0	0	2	1	0	0
Sutilizona theca	0	0	0	9	0	0	0	0	0	0	0	0	1	5	0	0	0	0	0	0	0	0	0	0	0	0	0
gastropods, unk	0	0	0	0	0	0	0	0	0	2	4	0	0	0	0	0	0	0	0	0	0	48	1	8	11	0	10
Bathymodiolus thermophilus	0	0	0	1	0	1	0	0	0	0	0	1	0	0	1	3	1	0	2	0	1	2	0	25	11	0	13
aplacophoran	0	0	0	0	0	0	0	0	0	0	0	0	0	0	0	0	0	0	0	0	0	0	0	1	0	1	0
Alvinella sp.	0	1	0	0	0	0	0	0	0	0	0	0	0	0	0	0	0	0	0	0	0	0	0	0	0	0	0
Amphisamytha galapagensis	0	0	0	87	29	7	0	0	0	20	5	14	2	0	2	1	12	0	2	1	0	85	82	172	39	8	47
Archinome rosacea	0	0	0	0	0	0	0	0	0	0	0	1	0	0	0	0	0	0	0	0	0	0	0	0	0	0	0
Branchinotogluma sp.	0	0	0	0	0	0	0	0	0	0	0	0	0	0	0	1	2	0	0	0	3	0	0	0	0	0	0
Branchiplicatus cupreus	0	0	0	0	0	0	0	0	0	0	0	0	0	0	0	0	5	0	2	1	1	0	0	0	0	0	0
Branchipolynoe sp.	0	0	0	0	0	0	0	0	0	0	0	0	0	0	0	0	0	0	0	0	0	0	0	0	0	0	1
Galapagomystides aristata	0	0	0	0	0	0	0	0	0	0	3	0	0	0	2	0	0	0	0	0	0	2	0	14	10	0	0
Glycera sp	0	0	0	0	0	0	0	0	0	0	0	0	0	0	1	0	0	0	0	0	0	0	0	0	0	0	0
Hesiospina vestimentifera	0	0	0	0	0	0	0	0	0	0	0	0	0	0	0	1	0	0	0	0	0	0	0	0	0	0	0
hesionid	0	0	0	0	0	0	0	0	0	0	0	0	3	0	7	0	0	0	0	0	0	0	0	0	0	0	0
Laminatubus alvini	0	0	0	0	0	0	0	0	0	0	0	0	0	0	0	0	0	0	0	0	0	0	0	9	0	0	1
Lepidonotopodium sp.	0	0	0	0	0	0	0	0	2	0	0	0	0	0	0	0	0	0	0	0	1	0	0	0	0	1	0
Nereis sp.	0	0	0	0	0	0	0	0	0	0	0	0	0	0	0	0	0	0	0	0	0	1	0	0	0	0	0
Ophryotrocha akessoni	0	0	0	39	36	89	0	0	0	27	14	17	10	26	30	68	224	21	52	12	34	19	16	18	18	6	10
Paralvinella grasslei	158	121	112	0	0	5	23	109	106	0	0	1	0	0	1	7	138	193	2	47	0	1	0	50	0	0	0
polynoids, unk	0	0	0	1	4	0	1	0	0	5	2	2	6	5	20	0	0	0	1	0	0	0	1	1	0	0	0
Prionospio sandersi	0	0	0	0	0	0	0	0	0	0	0	0	0	0	0	1	0	0	0	0	0	0	0	0	0	0	0
polychaetes, unk	0	0	0	54	17	68	0	1	0	0	1	2	0	0	0	0	0	0	0	0	0	0	0	0	1	0	0
Riftia pachyptila	0	0	0	0	0	0	0	0	0	0	0	0	0	0	0	2	0	0	0	0	0	0	2	4	0	0	3
Tevnia jerichonana	0	0	0	0	0	0	0	0	0	0	0	0	0	0	0	51	158	52	0	119	3	0	9	1	0	3	0
vestimentiferans, small	263	646	1087	149	318	1087	3	10	26	0	34	14	0	0	0	390	314	193	227	361	259	116	293	223	29	229	728
amphipod	0	1	1	0	1	4	28	1	6	14	58	35	2	1	5	938	427	24	334	285	918	24	3	20	5	15	17
Bythograea thermydron	2	3	3	0	1	0	4	1	2	2	1	1	0	0	0	1	4	0	2	4	1	0	0	0	0	0	0
Dahlella caldariensis	0	0	0	0	0	0	0	0	0	0	0	0	0	0	0	0	0	0	15	11	3	0	0	0	0	4	0
juv. shrimp	0	0	0	0	0	1	0	0	0	0	0	0	0	0	0	0	0	0	0	0	0	0	0	0	0	0	0
unsegmented worm	0	0	0	0	0	1	0	0	0	0	1	1	0	0	0	5	28	1	4	1	0	0	0	0	0	3	1
anemone	0	0	0	1	0	0	0	0	0	0	0	0	0	0	0	0	0	0	0	0	0	0	0	0	0	0	0
dandelion	0	1	0	0	0	0	0	0	0	0	0	0	0	0	0	0	0	0	0	0	0	0	0	0	0	0	0
kinorhynch	0	0	0	3	0	0	0	0	0	0	0	0	0	0	0	0	0	0	0	0	0	0	0	0	0	0	0
Abyssotherma pacifica	0	0	0	0	0	0	0	0	0	0	0	0	0	0	0	0	0	0	0	0	0	0	0	3	0	0	2
Metafolliculina sp.	0	0	0	0	0	0	0	0	0	0	0	0	0	0	0	0	0	0	0	0	0	0	0	0	0	1	2
