# Supplementary material for: Detecting the Influence of Initial Pioneers on Succession at Deep-Sea Vents
Source: PLoS One. 2012 Dec 4;7(12):e50015. doi: 10.1371/journal.pone.0050015 (PMC3514232; doi:10.1371/journal.pone.0050015)
Supplement: Table S3 — Analysis of variance (ANOVA) for Time. Species abundance was compared between colonization surfaces recovered at 9, 11 and 22 months after eruption from the hot environment at P-vent (SS = sum of squares, df = degrees of freedom, MS = mean squares). Data transformed as arcsine(square-root(relative abundance)). Post-hoc Tukey test used when P<0.05. Significant differences (bold) include Bonferroni correction for multiple tests, with significance level adjusted as appropriate for pioneer colonists (6 species, P<0.008) and later arrivals (6 species, P<0.008). T. jerichonana group includes small vestimentiferan tubeworms. (PDF) [file pone.0050015.s004.pdf]

Source	SS	df	MS	F	P	Tukey
Ctenopelta porifera	0.0616	2	0.0308	1.50	0.295	
Error	0.1228	6	0.0205			
Cyathermia naticoides	0.2926	2	0.1463	2.58	0.155	
Error	0.3399	6	0.0566			
Lepetodrilus tevnianus	0.2111	2	0.1056	1.15	0.378	
Error	0.5513	6	0.0919			
Paralvinella grasslei	0.4680	2	0.2340	5.60	0.043	11>22
Error	0.2509	6	0.0418			
Tevnia jerichonana	**0.9636**	**2**	**0.4818**	**32.68**	**0.001**	**9>22=11**
Error	0.0885	6	0.0147			
Bythograea thermydron	0.0205	2	0.0102	5.02	0.052	
Error	0.0122	6	0.0020			
Lepetodrilus elevatus	0.2046	2	0.1023	7.57	0.023	22>9=11
Error	0.0810	6	0.0135			
Rhynchopelta contentrica	0.0011	2	0.0006	3.97	0.080	
Error	0.0008	6	0.0001			
Bathymodiolus thermophilus	0.0009	2	0.0005	2.69	0.146	
Error	0.0010	6	0.0002			
Amphisamytha galapagensis	0.0019	2	0.0010	2.54	0.159	
Error	0.0023	6	0.0004			
Ophryotroch akessoni	**0.0990**	**2**	**0.0495**	**30.64**	**0.001**	**22>9=11**
Error	0.0097	6	0.0016			
Riftia pachyptila	0.0003	2	0.0002	1.00	0.422	
Error	0.0009	6	0.0002			
