# Supplementary material for: Detecting the Influence of Initial Pioneers on Succession at Deep-Sea Vents
Source: PLoS One. 2012 Dec 4;7(12):e50015. doi: 10.1371/journal.pone.0050015 (PMC3514232; doi:10.1371/journal.pone.0050015)
Supplement: Table S4 — Analysis of variance (ANOVA) for Disturbance. *ANOVA compromised by heteroscedasticity; Kruskal Wallis test significant P<0.005. Species abundance was compared between colonization surfaces recovered at sites with different disturbance histories: P-vent (post-eruption, disturbed), V-vent (post-eruption, undisturbed), and Pre-eruption. Data are transformed as arcsin(square-root(relative abundance)). Post-hoc Tukey test used when P<0.05. Significant differences (bold) include Bonferroni correction for multiple tests, with significance level adjusted as appropriate for pioneer colonists (6 species, P<0.008) and later arrivals (6 species, P<0.008). (PDF) [file pone.0050015.s005.pdf]

Source	SS	df	MS	F	P	Tukey
Ctenopelta porifera	0.0001	2	0.0000	1.93	0.195	
Error	0.0002	10	0.0000			
Cyathermia naticoides	0.0722	2	0.0361	3.83	0.058	
Error	0.0942	10	0.0094			
Lepetodrilus tevnianus	**0.0227**	**2**	**0.0114**	**7.71**	**0.009***	**P=V>pre**
Error	0.0147	10	0.0015			
Paralvinella grasslei	0.0118	2	0.0059	2.54	0.128	
Error	0.0232	10	0.0023			
Tevnia jerichonana	0.0045	2	0.0022	2.46	0.136	
Error	0.0091	10	0.0009			
Bythograea thermydron	**0.0000**	**2**	**0.0000**	**9.65**	**0.005**	**V > P>pre**
Error	0.0000	10	0.0000			
Lepetodrilus elevatus	0.2304	2	0.1152	3.67	0.064	
Error	0.3141	10	0.0314			
Amphisamytha galapagensis	0.0316	2	0.0158	5.19	0.028	Pre>V=P
Error	0.0304	10	0.0030			
Ophryotrocha akessoni	0.0008	2	0.0004	0.88	0.443	
Error	0.0046	10	0.0005			
Lepetodrilus pustulosus	0.006	2	0.0030	1.89	0.202	
	0.016	10	0.0016			
Bathymodiolus thermophilus	0.0002	2	0.0001	2.17	0.164	
Error	0.0006	10	0.0001			
Riftia pachyptila	0.0000	2	0.0000	0.93	0.426	
Error	0.0003	10	0.0000			
