# Supplementary material for: Detecting the Influence of Initial Pioneers on Succession at Deep-Sea Vents
Source: PLoS One. 2012 Dec 4;7(12):e50015. doi: 10.1371/journal.pone.0050015 (PMC3514232; doi:10.1371/journal.pone.0050015)
Supplement: Table S5 — Analysis of variance (ANOVA) for Environment at 11 months. *Probably Tevnia jerichonana. Species abundance was compared across different thermal environments (hot, warm, cool) at P-vent, 11 mo after eruption. Data are transformed ln(abundance)+1). Post-hoc Tukey test used when P<0.05. Significant differences (bold) include Bonferroni correction for multiple tests, with significance level adjusted for pioneer colonists (6 species, P<0.008) and later arrivals (5 species, P<0.01). (PDF) [file pone.0050015.s006.pdf]

Source	SS	df	MS	F	P	Tukey
Ctenopelta porifera	8.648	2	4.324	3.86	0.084	
Error	6.726	6	1.121			
Cyathermia naticoides	1.854	2	0.927	2.12	0.201	
Error	2.624	6	0.437			
Lepetodrilus tevnianus	26.716	2	13.358	5.41	0.045	W>H
Error	14.821	6	2.470			
Paralvinella grasslei	**31.248**	**2**	**15.624**	**43.44**	**<0.001**	**H>W=C**
**Error**	**2.158**	**6**	**0.360**			
*Siboglinid tubeworms, small	10.003	2	5.001	3.44	0.101	
Error	8.723	6	1.454			
Bythograea thermydron	**2.065**	**2**	**1.032**	**11.66**	**0.009**	**H=W>C**
**Error**	**0.531**	**6**	**0.089**			
Amphisamytha galapagensis	**10.037**	**2**	**5.019**	**18.30**	**0.003**	**W>C=H**
**Error**	**1.645**	**6**	**0.274**			
Ophryotrocha akessoni	**18.123**	**2**	**9.062**	**64.81**	**<0.001**	**C=W>H**
**Error**	**0.839**	**6**	**0.140**			
Bathymodiolus thermophilus	0.107	2	0.053	0.50	0.630	
Error	0.641	6	0.093			
Lepetodrilus pustulosus	**4.713**	**2**	**2.356**	**10.85**	**0.010**	**W>H=C**
**Error**	**1.303**	**6**	**0.217**			
**Polynoid**	**6.193**	**2**	**3.097**	**11.80**	**0.008**	**C>H**
**Error**	**1.574**	**6**	**0.262**			
