# Supplementary material for: Detecting the Influence of Initial Pioneers on Succession at Deep-Sea Vents
Source: PLoS One. 2012 Dec 4;7(12):e50015. doi: 10.1371/journal.pone.0050015 (PMC3514232; doi:10.1371/journal.pone.0050015)
Supplement: Table S6 — Analysis of variance (ANOVA) for Environment at 9 months. *Probably Tevnia jerichonana. Species abundance was compared between colonization surfaces recovered from different thermal environments (hot at P-vent and warm at Ty/Io), 9 mo after eruption. Data are transformed ln(abundance+1). Post-hoc Tukey test used when P<0.05. Significant differences (bold) include Bonferroni correction for multiple tests, with significance level adjusted as appropriate for pioneer colonists (6 species, P<0.008) and later arrivals (5 species, P<0.01). (PDF) [file pone.0050015.s007.pdf]

Source	SS	df	MS	F	P	Tukey
Ctenopelta porifera	**16.548**	**1**	**16.548**	**49.14**	**0.002**	**H>W**
Error	1.347	4	0.337			
Cyathermia naticoides	2.701	1	2.701	7.90	0.048	
Error	1.367	4	0.342			
Lepetodrilus tevnianus	**97.759**	**1**	**97.759**	**60.83**	**0.001**	**W>H**
Error	6.429	4	1.607			
Paralvinella grasslei	**44.783**	**1**	**44.783**	**81.26**	**0.001**	**H>W**
Error	2.204	4	0.551			
***Siboglinid tubeworms (small)**	**225.809**	**1**	**225.809**	**298.43**	**<0.001**	**H>W**
Error	3.027	4	0.757			
Bythograea thermydron	**3.472**	**1**	**3.472**	**36.99**	**0.004**	**H>W**
Error	0.375	4	0.094			
Amphisamytha galapagensis	**16.527**	**1**	**16.527**	**22.91**	**0.009**	W>H
Error	2.885	4	0.721			
Ophryotroch akessoni	**23.205**	**1**	**23.205**	**191.54**	**<0.001**	**W>H**
Error	0.485	4	0.121			
Bathymodiolus thermophilus	0.320	1	0.320	4.00	0.116	
Error	0.320	4	0.080			
Clypeosectus delectus	0.432	1	0.432	1.00	0.374	
Error	1.727	4	0.432			
Polynoid	0.884	1	0.884	2.71	0.175	
Error	1.303	4	0.326			
